# Increased Dopamine Type 2 Gene Expression in the Dorsal Striatum in Individuals With Autism Spectrum Disorder Suggests Alterations in Indirect Pathway Signaling and Circuitry

**DOI:** 10.3389/fncel.2020.577858

**Published:** 2020-11-09

**Authors:** Cheryl Brandenburg, Jean-Jacques Soghomonian, Kunzhong Zhang, Ina Sulkaj, Brianna Randolph, Marissa Kachadoorian, Gene J. Blatt

**Affiliations:** ^1^Autism Neurocircuitry Laboratory, Hussman Institute for Autism, Baltimore, MD, United States; ^2^Program in Neuroscience, University of Maryland Baltimore School of Medicine, Baltimore, MD, United States; ^3^Department of Anatomy and Neurobiology, Boston University School of Medicine, Boston, MA, United States

**Keywords:** autism spectrum disorder, dopamine, GABA, basal ganglia, indirect pathway, risperidone

## Abstract

Autism spectrum disorder (ASD) is behaviorally defined and diagnosed by delayed and/or impeded language, stereotyped repetitive behaviors, and difficulties with social interactions. Additionally, there are disruptions in motor processing, which includes the intent to execute movements, interrupted/inhibited action chain sequences, impaired execution of speech, and repetitive motor behaviors. Cortical loops through basal ganglia (BG) structures are known to play critical roles in the typical functioning of these actions. Specifically, corticostriate projections to the dorsal striatum (caudate and putamen) convey abundant input from motor, cognitive and limbic cortices and subsequently project to other BG structures. Excitatory dopamine (DA) type 1 receptors are predominantly expressed on GABAergic medium spiny neurons (MSNs) in the dorsal striatum as part of the “direct pathway” to GPi and SNpr whereas inhibitory DA type 2 receptors are predominantly expressed on MSNs that primarily project to GPe. This study aimed to better understand how this circuitry may be altered in ASD, especially concerning the neurochemical modulation of GABAergic MSNs within the two major BG pathways. We utilized two classical methods to analyze the postmortem BG in ASD in comparison to neurotypical cases: ligand binding autoradiography to quantify densities of GABA-A, GABA-B, 5-HT_2_, and DA type 1 and 2 receptors and *in situ* hybridization histochemistry (ISHH) to quantify mRNA for D1, D2 receptors and three key GABAergic subunits (α1, β2, and γ2), as well as the GABA synthesizing enzymes (GAD65/67). Results demonstrated significant increases in D2 mRNA within MSNs in both the caudate and putamen, which was further verified by proenkephalin mRNA that is co-expressed with the D2 receptor in the indirect pathway MSNs. In contrast, all other GABAergic, serotonergic and dopaminergic markers in the dorsal striatum had comparable labeling densities. These results indicate alterations in the indirect pathway of the BG, with possible implications for the execution of competing motor programs and E/I imbalance in the direct/indirect motor feedback pathways through thalamic and motor cortical areas. Results also provide insights regarding the efficacy of FDA-approved drugs used to treat individuals with ASD acting on specific DA and 5-HT receptor subtypes.

## Introduction

Characteristic delayed and/or impeded language, stereotyped repetitive behaviors, and difficulties with social interaction/communication are the core features of an autism spectrum disorder (ASD) diagnosis (Geschwind and Levitt, 2007; Fung and Hardan, 2014). Additionally, motor impairment is a cardinal feature of ASD, and language to reflect repetitive motor behaviors has been added to the DSM-5 diagnostic criteria (American Psychiatric Association, 2013). The behaviors associated with disrupted motor processing are widespread and include differences in fundamental motor skills such as eye movement, fine and gross motor skills, gait and balance as well as more complex skills like movement coordination, action chaining, and inhibition control (for a review see: Becker and Stoodley, 2013; Subramanian et al., 2017). Given the clear disruption in sensorimotor processing in individuals with ASD, it is important to examine postmortem brain areas that are responsible for the intention to execute movements.

The basal ganglia (BG) are known to participate in action selection, learned habits, action sequences, and repetitive behaviors (for a review see: Graybiel, 2008; Graybiel and Grafton, 2015) and increasing evidence implicates the BG in the pathogenesis of ASD. Several studies have found volumetric differences in the dorsal striatum (caudate and/or putamen) of ASD subjects compared to neurotypical individuals *via* imaging studies (Sears et al., 1999; Hollander et al., 2005; Rojas et al., 2006; Langen et al., 2007; Sato et al., 2014) and one postmortem study demonstrated similar findings (Wegiel et al., 2014). Thus, neuroanatomical differences in the dorsal striatum in individuals with ASD suggest that critical loops within the BG and their cortical connections may be significantly impacted. Increased caudate volume in ASD (Sears et al., 1999; Hollander et al., 2005; Rojas et al., 2006; Wegiel et al., 2014), for example, has been related to complex mannerisms, compulsions/rituals, stereotypy and/or difficulties in routine scores on the Autism Diagnostic Interview (ADI).

Afferent input from the frontal and cingulate cortices to the dorsal striatum provides motor, limbic, and cognitive information (for a review see: Subramanian et al., 2017) and may participate in ASD-related functions. Glutamatergic inputs provide excitatory drive from the thalamus and cortex and mostly target GABAergic medium spiny neurons (MSNs) and GABAergic interneurons, which, *via* feed-forward synapses, inhibit MSNs, which in turn primarily project to the globus pallidus internus (GPi) or substantia nigra pars reticularis (SNpr; direct pathway; rich in D1 receptors) or the globus pallidus externus (GPe; indirect pathway; rich in D2 receptors). The interplay of these pathways likely affects action performance by facilitation of the selection of action and inhibiting unwanted actions following cortical activation (Lovinger, 2017).

Despite increased recognition that the BG is implicated in ASD, there is a wide gap in our knowledge regarding which aspects of the BG circuitry are impacted in ASD as well as a lack of understanding of defined targets for pharmacotherapeutic treatment. Interestingly, the first drug approved for children with ASD and now the most widely used, Risperidone (Risperdal), has led to significant improvements in behavioral symptoms, such as sensorimotor and repetitive behaviors (McCracken et al., 2002; Shea et al., 2004; McDougle et al., 2005; Pandina et al., 2007; Kent et al., 2013; Goel et al., 2018). Risperidone mainly has high-affinity binding as an antagonist at serotonin (5-HT) 2A receptors and dopamine (DA) type 2 receptors (D2R). Aripiprazole (Abilify), one of the very few other drugs approved by the FDA for ASD (LeClerc and Easley, 2015), acts as a partial agonist to D2R and 5-HT_1A_ receptors and as an antagonist to 5-HT_2A_ receptors (Hirsch and Pringsheim, 2016; Lamy and Erickson, 2018), but targets anxiolytic symptoms (Marcus et al., 2009; Owen et al., 2009; Ichikawa et al., 2017). Although there are clinical reports for use of both of these drugs for ASD patients, there are no postmortem studies from ASD subjects that quantify the density and distribution of DA and 5-HT receptors in key brain structures that are likely involved in repetitive behaviors, such as the BG.

The objective of the current study was to determine if levels of expression of markers of dopaminergic, GABAergic, and serotonergic activity in the striatum are changed in ASD, which could shed light on the mechanisms whereby the BG is impacted. This study utilizes classic methodologies such as *in situ* hybridization histochemistry (ISHH) and ligand binding autoradiography to quantify select GAD/GABA receptor and DA receptor mRNA expression as well as to quantify GABA, DA, and 5-HT receptor densities in the dorsal striatum of postmortem ASD cases as compared to controls. These methodologies were designed to determine whether there are alterations within the circuitry of the dorsal striatum and/or in receptor density, including those which are primary targets for risperidone and aripiprazole, within the corticostriatal and striatopallidal circuits in age, postmortem interval (PMI), and gender-matched cases. Collectively, these data help to improve our understanding of neurochemical differences within the BG that can inform on likely targets responsible for dysregulation of motor behaviors in ASD. The results indicate a largely normal repertoire of DA, GABA and 5-HT receptors expressed within the dorsal striatum, but reveal an increase of Drd2 mRNA expression within individual MSNs that likely impacts output to the GPe. These insights help to clarify which of the key targets for approved and widely used ASD drugs are altered in the BG of a cohort of postmortem ASD cases.

## Materials and Methods

### Postmortem Tissue

Human postmortem brain tissue was obtained from the University of Maryland Brain and Tissue Bank, a brain and tissue repository of the NIH Neurobiobank. Case demographics are detailed in Table 1. Coronal sections (20 μm) from caudate and putamen were cut on a Leica CM1950 cryostat and kept frozen at −80°C (*n* = 11 control, *n* = 11 ASD). Note that brain blocks through a portion of the dorsal striatum were matched for level as far as having both caudate and putamen present in the same sections when cut, but the exact level matching of individual sections is a limitation in postmortem studies due to the availability of material through regions of interest. Several blocks did not contain ventral striatum. The number of cases used in individual experiments may vary due to the occasional loss of sections during processing. Total case ages (*p* = 0.24) and PMI (*p* = 0.37) were not significantly different between ASD and control cases using a Student’s *t*-test. Three ASD cases had at least one seizure reported and six had reported medications (Table 1). All ASD cases had confirmed diagnoses through the Autism Diagnostic Interview-Revised (ADI-R) scores and/or received a clinical diagnosis of autism from a licensed psychiatrist.

**Table 1:** Postmortem brain donor case demographics.

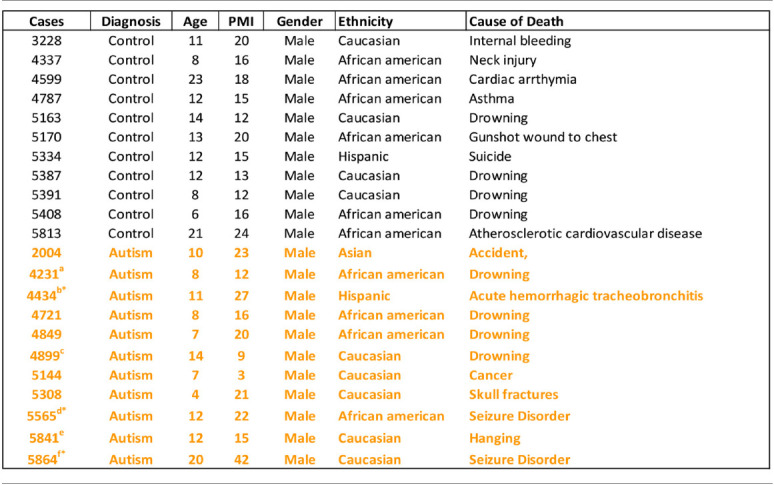

*At least one documented seizureKnown medications: ^a^Zyprexa, Lexapro; ^b^Keppra, Lexapro; ^c^Keppra; ^d^Daytrana; ^e^Trileptal, Zoloft, Clonidine, Melatonin; ^f^Tegretol, Clonidine, Ativan, Risperdal.

As this research did not involve live human subjects, Institutional Review Board approval and informed consent were not necessary. However, the University of Maryland Brain and Tissue Bank (NIH Neurobiobank) is overseen by Institutional Review Board protocol number HM-HP-00042077 and de-identifies all cases before distribution to researchers.

### Radioisotopic *In Situ* Hybridization Histochemistry (ISHH)

^35^S radiolabeled complementary RNA (cRNA) probes were transcribed *in vitro* from cDNAs selective for the human GAD67, GAD65, PPE, dopamine Drd2, and Drd1 receptors and GABAa alpha1, beta2, and gamma 2 receptor subunits. The circular plasmids containing the cDNAs were linearized according to standard protocols (Wood, 1983). Transcription of the radioactive cRNAs was performed for 2 h at 37°C in the presence of 2.5 μM ^35^S-uracil triphosphate (UTP; specific activity 1,250 Ci/mmol; Perkin Elmer Life Sciences) and 10 μM unlabeled UTP with ATP, cytosine triphosphate (CTP), and guanine triphosphate (GTP) in excess. The cDNA template was then digested with DNAse I. The labeled cRNAs were purified by phenol/chloroform extraction and ethanol precipitation and the probe length was reduced to 100–150 nucleotides by alkaline hydrolysis (Cox et al., 1984).

Two adjacent sections per subject were used. Sections were fixed for 5 min in 3% paraformaldehyde in 0.1 M phosphate buffer saline (pH 7.2). Pre-hybridization washes were in 2× SSC, phosphate buffer saline (0.4 M), 0.25% acetic anhydride with triethanolamine, and Tris-glycine, then followed by dehydration in ethanol. After rinsing with 2× SSC, sections hybridized for 4 h at 52°C with 8 ng of radiolabeled cRNA probe. The probe was diluted in 20 μl of hybridization solution (containing 40% formamide, 10% dextran sulfate, 4× SSC, 10 mM dithiothreitol, 1.0% sheared salmon sperm DNA, 1.0% yeast tRNA, and 1× Denhardt’s solution). The sections were subsequently washed in 50% formamide at 52°C for 5 and 20 min, RNAse A (100 μg/ml; Sigma–Aldrich) for 30 min at 37°C, and in 50% formamide for 5 min at 52°C, then dehydrated in ethanol and defatted in xylene. Sections were placed in contact with Kodak BioMax MR film in light-tight cassettes for 10–15 days. The films were then developed. Slides were then processed for emulsion autoradiography. In that case, slides were coated with Kodak NTB3 nuclear emulsion diluted 1:1 with distilled water containing 300 mM ammonium acetate, air-dried for 3 h, and stored at room temperature in light-tight boxes for 14 days. Sections were developed in Kodak D-19 developer for 3.5 min at 14°C and lightly counterstained with eosin and hematoxylin, and mounted with Eukitt.

### Quantification of mRNA Labeling

The relative levels of GAD67, GAD65, PPE, Drd2, Drd1, α1, β2, and γ2 mRNA labeling were quantified on X-ray film autoradiographs by computerized densitometry with NIH Image (Macintosh^1^). The autoradiographs were digitized using a CCD Sony video camera and the analog signal was converted to a digital image of 640 × 480 pixels (picture points) with gray values ranging from 0 to 255. Quantification was conducted ensuring most values were in the linear range of the best-fit calibration curve. Relative optical density (OD) measurements were calculated using two adjacent slides from each case. Data were expressed as mean ± S.E.M. Relative differences were compared within groups by Student’s *t*-test.

Following processing with X-ray films, sections were processed for emulsion autoradiography by dipping in Kodak NTB liquid emulsion. Following 2–3 weeks of exposure duration, the emulsion autoradiographs were developed in Kodak D19 developer, processed in fixative and counterstained with eosin-hematoxylin. Following mounting, the autoradiographs were examined on a Nikon microscope and neurons with five or more silver grains were quantified using NIH image as previously described (Nielsen and Soghomonian, 2004; Lanoue et al., 2010). Only probes showing a significant effect on X-ray films were quantified on emulsion autoradiographs. Two sections per case and 50 neurons per section and per region (caudate and putamen) were analyzed. The results were expressed as a number of pixels per neuron. Data from control and ASD cases were plotted for the caudate and the putamen as a frequency distribution of labeling per neuron and comparisons between the distributions in controls and ASD cases were analyzed with a Kolmogorov-Smirnov test.

### Saturation Ligand Binding Assays

Five tritiated [^3^H] ligands including spiperone [Perkin Elmer, Boston, MA, USA, NET1187 (2016); Palacios et al., 1981; Filer et al., 2019], SCH 23390 [NET930 (2015); Bourne, 2001], flunitrazepam [NET5627 (2015); Guptill et al., 2007; Oblak et al., 2009], Ketanserin [NET7910 (2015); Leysen et al., 1982] and CGP 54626 [American Radiolabeled Chemicals, St. Louis, MO, USA ART715 (2015); Scheperjans et al., 2005] were processed under conditions specified in Table 2. Total binding was quantified from two thawed 20 μm sections while one section from each case was exposed to the ligand and a displacer to determine non-specific binding. Each displacer purchased from Sigma–Aldrich (St. Louis, MO, USA) was used at a concentration of 10 μM for +/− butaclamol hydrochloride [SIG-D033 (2016)] or 100 μM for both Clonazepam [SIG-C1277 (2016)] and CGP-55845 hydrochloride [SIG-SML0594 (2015)]. Imipramine hydrochloride was also used at 100 μM [AAJ6372306 Thermo Fisher Scientific, Waltham, MA, USA (2016)]. Labetalol hydrochloride [SIG L1011 (2016)] and ketanserin +/− tartrate [SIG S006 (2016)] were added to the spiperone buffer to block serotonin receptor 2 and adrenoreceptors.

**Table 2:** Ligand binding conditions.

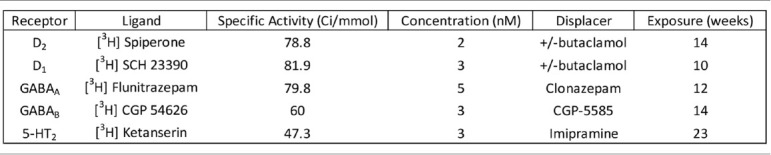

All tissue went through a pre-incubation in buffer without ligand for 30 min before incubation with a ligand for 1 h. Buffer for ^3^H spiperone was composed of 50 mM Tris-HCl, 120 mM NaCl, 5 mM KCl, 2 mM CaCl_2_, 1 mM MgCl_2_ pH 7.4, ^3^H SCH 23390 and ^3^H flunitrazepam was composed of 170 mM Tris-HCl, pH 7.4 and ^3^H CGP 54626 used 50 mM Tris-HCl and 2.5 mM CaCl_2_, pH 7.2. After three 5-min rinses in buffer followed by one dip in distilled water, sections were allowed to air-dry overnight. Slides, [^3^H]-sensitive hyper film [Kodak Biomax MR film Z350389, Sigma–Aldrich, St. Louis, MO, USA (2016)] and a [^3^H] standard [Tritium standards, American Radiolabeled Chemicals St. Louis, MO, USA ART0123 (2015)] were placed in X-ray cassettes and exposed for 10–14 weeks. Films were processed in the dark for 3 min in developer [Kodak D19 74200, Electron Microscopy Sciences, Hatfield, PA, USA (2015)], fixed [Kodak Rapidfix 74312, Electron Microscopy Sciences, Hatfield, PA, USA (2015)] for 4 min at room temperature, and washed with a stream of water for 1 h and air-dried.

**Figure 1:**
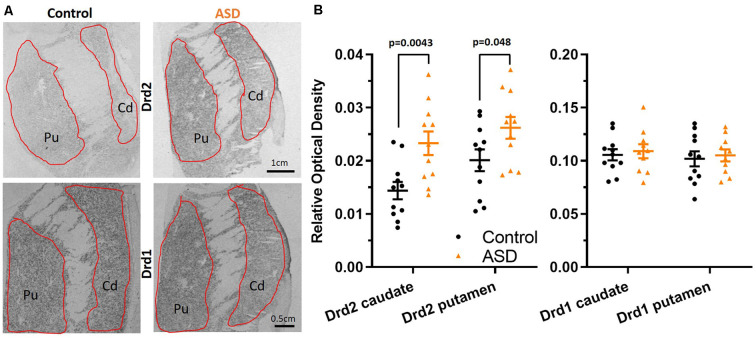
**(A)** Radioisotopic *in situ* hybridization histochemistry showing labeled mRNA of Drd2 and Drd1. **(B)** Quantification of the relative density of Drd2 measured on film autoradiographs reveals higher expression in autism spectrum disorder (ASD) cases (orange) in both the caudate and putamen. Drd1 expression levels are unchanged in the caudate and putamen in ASD. Drd2 *n* = 11 control, 11 ASD; Drd1 *n* = 11 control, 10 ASD.

Tissue section autoradiograms from the film were digitized with a QICAM digital camera (QImaging, Surrey, BC, Canada) followed by analysis with MCID Core 7.1 Elite Image analysis system software (InterFocus Imaging Limited, UK). Within the MCID software, one representative standard curve was chosen to normalize all standard curves and images for comparison across films. The ribbon tool was employed to sample binding along the length of the observable region of interest that included the caudate and putamen of the BG (as shown by the Nissl in Figure 1A). OD values of the sampled areas were converted to nanocuries (nCi) per milligram (mg) through the use of [^3^H] standards then femtomoles (fmol) per mg of protein-based on the specific activity for each ligand. As non-specific binding was less than 5% of total binding, total binding was taken as representative of the specific binding for each ligand. Student *t*-tests were conducted to compare the binding affinity of each ligand between control and ASD groups.

## Results

### Dopamine D1 and D2 Receptor Analysis

Radioisotopic ISHH allowed for visualization of mRNA encoding for dopamine receptors (Drd2, Drd1) within the caudate and putamen (Figure 1A). Regional quantification on film autoradiographs indicated that Drd2 mRNA expression was significantly elevated in both the caudate (mean: control 0.014 ± 0.002, ASD 0.023 ± 0.002) and putamen (mean: control 0.020 ± 0.002, ASD 0.026 ± 0.002) of the ASD cases (Figure 1B), with a more pronounced difference shown in the caudate. The expression of the Drd1 mRNA was not different between controls and ASD for neither the caudate (mean: control 0.106 ± 0.005, ASD 0.109 ± 0.007) or putamen (mean: control 0.102 ± 0.007, ASD 0.105 ± 0.006; Figure 1B).

**Figure 2:**
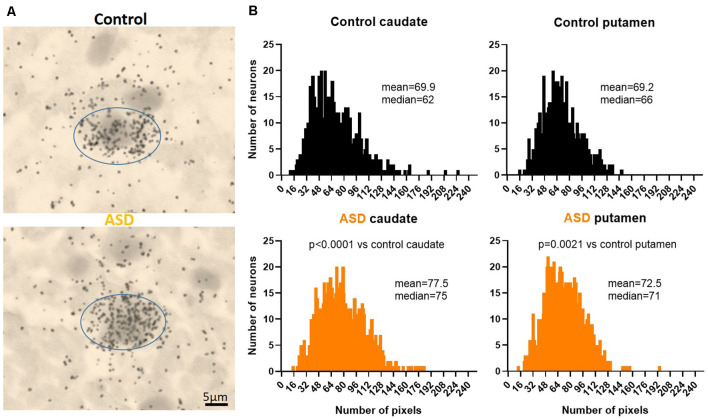
**(A)** Representative images of control and ASD caudate. Drd2 emulsion autoradiographs. **(B)** Histograms of Drd2 mRNA labeling within individuals cells quantifies the distribution from emulsion autoradiographs in controls (upper panels) and ASD (lower panels). Differences between control and ASD distributions were analyzed with a Kolmogorov–Smirnov test. *n* = 10 control, 11 ASD cases; *n* = 884 control caudate, 1,004 autism caudate, 766 control putamen, 1,000 autism putamen cells.

To further examine the increase in Drd2 expression, mRNA levels were quantified at the single-cell level on emulsion autoradiographs (Figure 2). The analysis yielded results that confirmed the effects seen on film autoradiographs and further indicated that higher Drd2 mRNA levels in ASD seen on X-ray films are due to an increased expression per neuron rather than an increase in the number of neurons expressing the mRNA.

**Figure 3:**
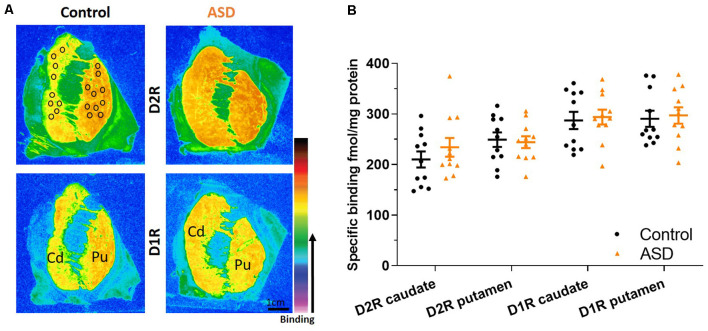
**(A)** Representative images of control and ASD cases for ligand binding with warmer colors indicating higher binding. Black circles over the control case represent typical sampling areas, where we chose only representative areas with appropriate binding levels for quantification and can be applied to all binding images. **(B)** D2 and D1 receptor expression labeled with tritiated isotopes are similar between ASD (orange) and controls (black) in the caudate/putamen. *n* = 11 control, 11 ASD.

To determine whether increased Drd2 mRNA levels were paralleled by increased receptor expression at the protein level, ligand binding assays were conducted. D2R binding levels within the caudate (mean: control 210.014 ± 15.946, ASD 234.108 ± 18.529) and putamen (mean: control 249.161 ± 14.201, ASD 244.190 ± 11.614) were similar between ASD and control cases (Figure 3), indicating that overall D2R tissue expression within the caudate and putamen is unchanged. The D2Rs are primarily distributed on indirect striatal pathway neurons, on cholinergic and GABAergic interneurons as well as on corticostriatal axonal projections, whereas the Drd2 mRNA is detected in intrinsic striatal neurons only. Also, the population of cholinergic interneurons is much lower than the population of indirect pathway neurons. One explanation for the contradictory results between ISHH and ligand binding experiments could be that the ISHH results primarily reflect a change in mRNA levels in indirect pathway neurons. On the other hand, potential changes in ligand binding in indirect pathway neurons may be obscured by the detection of receptors on other populations of neurons, including corticostriatal axons.

**Figure 4:**
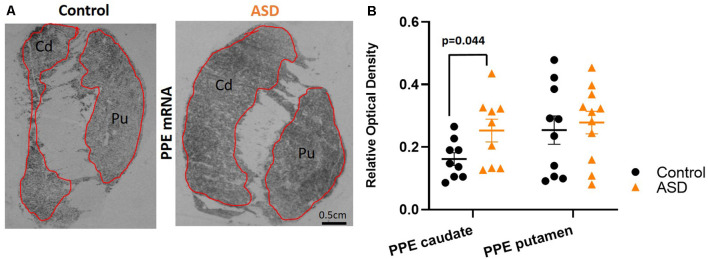
**(A)** Preproenkephalin mRNA labeling with radioisotopic *in situ* hybridization histochemistry. **(B)** Preproenkephalin is increased in the caudate, but not in the putamen of ASD cases. *n* = 10 control putamen, nine control caudate; 11 ASD putamen, nine ASD caudate.

To further assess the possibility that the expression of other mRNAs is altered in the indirect pathway neurons, we also measured pre proenkephalin (PPE; Figure 4A), which is selectively expressed in indirect pathway striatal neurons. Although the putamen levels of PPE in ASD cases were not different (mean: control 0.254 ± 0.045, ASD 0.278 ± 0.036; Figure 4B), the caudate of ASD had significantly higher levels of PPE compared to controls (mean: control 0.162 ± 0.020, ASD 0.253 ± 0.036).

### GABAergic and 5-HT Markers

**Figure 5:**
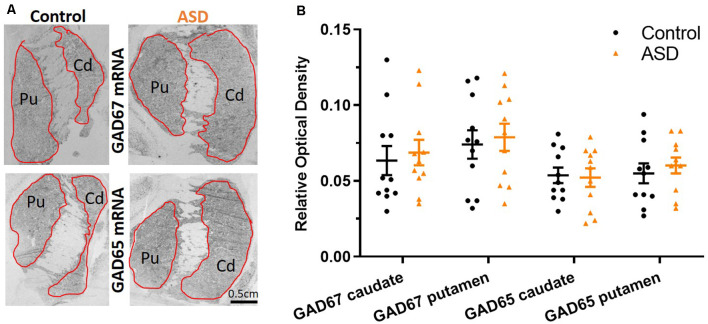
**(A)** GAD67 and GAD65 mRNA labeling with radioisotopic *in situ* hybridization histochemistry. **(B)** Both GAD65 and GAD67 are similarly expressed between ASD and control cases in the caudate and putamen. *n* = 11 control, 11 ASD.

**Figure 6:**
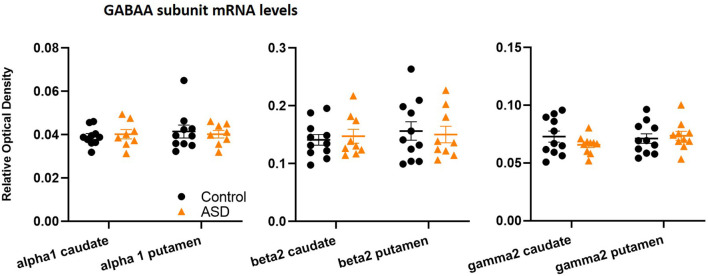
GABA_A_ subunit mRNA levels are unchanged between control and ASD cases, including α1 (*n* = 10 control, 8 ASD), β2 (*n* = 11 control, 9 ASD), and γ2 (*n* = 11 control, 10 ASD) in both caudate and putamen.

**Figure 7:**
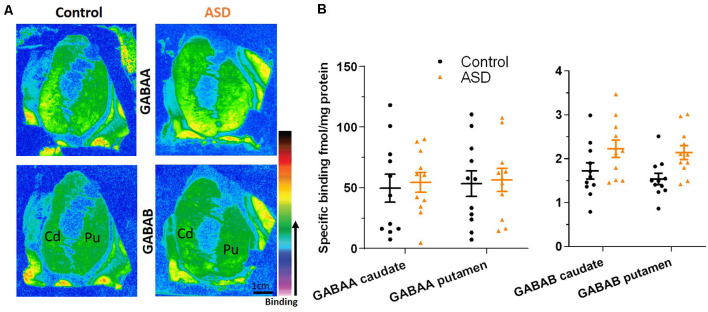
**(A)** Representative image of ligand binding with warmer colors indicating higher binding. **(B)** GABA_A_ and GABA_B_ receptor expression labeled with tritiated isotopes is similar between ASD (orange) and controls (black). *n* = 11 control, 11 ASD.

**Figure 8:**
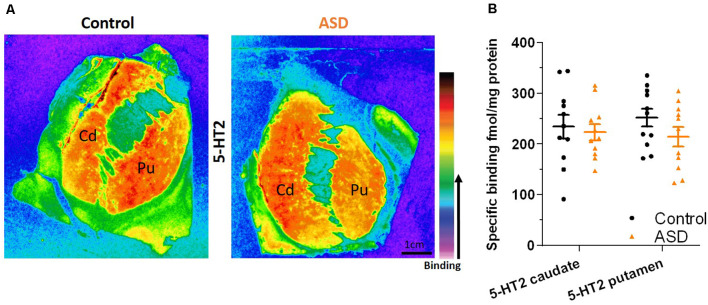
**(A)** Representative image of ligand binding with warmer colors indicating higher binding. **(B)** 5-HT_2_ receptor expression labeled with a tritiated isotope is similar between ASD (orange) and controls (black). *n* = 11 control, 11 ASD.

The mRNA levels of GAD65 caudate (mean: control 0.054 ± 0.005, ASD 0.052 ± 0.006) and putamen (mean: control 0.055 ± 0.007, ASD 0.060 ± 0.005) and GAD67 (Figure 5) in the caudate (mean: control 0.063 ± 0.010, ASD 0.069 ± 0.008) and putamen (mean: control 0.074 ± 0.009, ASD 0.079 ± 0.009) as well as the three GABA-A receptor subunits [α1 caudate (mean: control 0.039 ± 0.001, ASD 0.040 ± 0.002), putamen (mean: control 0.041 ± 0.003, ASD 0.040 ± 0.002); β2 caudate (mean: control 0.141 ± 0.009, ASD 0.147 ± 0.012), putamen (mean: control 0.156 ± 0.016, ASD 0.150 ± 0.014); and γ2 caudate (mean: control 0.073 ± 0.005, ASD 0.066 ± 0.002), putamen (mean: control 0.071 ± 0.004, ASD 0.074 ± 0.004); Figure 6] were not significantly different between control and ASD cases. Protein levels were not different between ASD and control groups for GABA_A_ [caudate (mean: control 49.688 ± 11.639, ASD 54.462 ± 8.060), putamen (mean: control 53.421 ± 10.626, ASD 56.352 ± 9.418)] and GABA_B_ receptors [caudate (mean: control 1.723 ± 0.184, ASD 2.229 ± 0.198), putamen (mean: control 1.540 ± 0.128, ASD 2.141 ± 0.159); Figure 7] or 5-HT_2_ receptors [caudate (mean: control 234.358 ± 23.548, ASD 223.460 ± 16.143), putamen (mean: control 251.705 ± 17.497, ASD 214.465 ± 19.519); Figure 8].

## Discussion

### Dopaminergic Expression Differences Are Evident in the Dorsal Striatum in ASD

The objective of the present study was to document possible changes in the expression of key markers of dopaminergic and GABAergic neurons in ASD. Our study presents original evidence for neurochemical imbalance in the dopaminergic system in the caudate and putamen in ASD cases. The functional relevance of differences in the BG of individuals with ASD is unclear but the effect seen on Drd2 receptors suggests that modulation of striatal neurons by dopamine is altered in ASD. Dopamine is a major modulator of striatal neurons and dopamine receptors are expressed in striatal interneurons and striatal projection neurons. Projection neurons in particular constitute a large proportion of the striatal neuronal population representing about 85% of all neurons. Other cells are cholinergic or GABAergic interneurons (Assous and Tepper, 2019). In our ISHH studies, analyses did not distinguish between GABAergic projection neurons and interneurons. Because the population of interneurons is very low compared to projection neurons, the changes in Drd2 mRNA documented here likely occurred in GABAergic projection neurons. Also, we did not include large cell bodies in our analyses, which correspond to cholinergic interneurons. It was somewhat surprising to find that the changes in Drd2 mRNA levels were not accompanied by changes in dopamine D2 receptors in our binding experiments. There are several plausible explanations for this apparent mismatch. First, ligand binding detects receptors present not only on projection neurons and interneurons but also on axon terminals of corticostriatal and thalamostriatal inputs. Second, dopamine receptors expressed in striatal projection neurons can be transported to their targets in the globus pallidus and/or substantia nigra, and changes in mRNA levels may not necessarily translate into changes in receptor levels on cell bodies. D2Rs expressed in striatal projection neurons are primarily expressed in indirect pathway neurons that project to the Gpe. This subpopulation of striatal projection neurons co-expressed PPE mRNA (for a review see: Soghomonian, 2016). We found that PPE mRNA levels were also elevated in the caudate, which further supports the interpretation that indirect pathway neurons are impacted in ASD. Drd1 mRNA levels, which are primarily expressed in the direct pathway neurons that project to Gpi were not changed.

Dopamine receptors exert widespread effects on the activity of intrinsic neurons in the caudate and putamen. In particular, Drd2 receptors inhibit striatal indirect pathway neurons. The changes in Drd2 mRNA levels suggest that control of GABAergic indirect pathway projection neurons is affected in ASD, however, a functional change in the striatum is undetermined and there may instead be an impact on D2R in the globus pallidus. A recent study has shown that a DAT mutation identified in an individual with ASD alters dopaminergic transmission in the striatum of a mouse model, an effect paralleled by hyperactive and repetitive behaviors as well as social deficits (DiCarlo et al., 2019). In this context, it would also be important to determine if changes in dopaminergic activity are a developmental feature of ASD. In that regard, it is noteworthy that gene mutations seen in ASD alter corticostriatal activity in mouse models (for a review see: Subramanian et al., 2017). In particular, in Fmr1 mice, a hypoconnectivity of corticostriatal synapses has been documented (Centonze et al., 2008; Jung et al., 2012; Zerbi et al., 2018). Whether or not dopamine plays a role in this hypoconnectivity would be important to determine. The reason why we did not see a parallel effect on GAD or GABA_A_ receptor expression is unclear. The most likely possibility is that GAD and GABA_A_ receptors are expressed in direct and indirect pathway neurons as well as in striatal interneurons. Therefore, a specific effect in indirect pathway neurons would be diluted. Future double-labeling anatomical studies at the single-cell level or single-cell transcriptomics could be used to determine if transcriptional changes are selective to indirect pathway neurons and if they involve other markers of activity.

### Corticostriatal Inputs to MSNs of the Direct and Indirect Pathways Are Modulated by Dopamine

The caudate and putamen receive widely distributed inputs from the cortex. These corticostriatal projections originate from cortical layers 5A, 5B, and 6 (for a review see: Kuo and Liu, 2019). Averbeck et al. (2014) used anterograde or bidirectional tract tracers in male macaque monkeys to map corticostriatal inputs, which are topographically organized but largely overlapping, especially from frontal cortical areas (for a review see: Haber, 2016). As an example, ventromedial prefrontal cortex (vmPFC), orbitofrontal cortex (OFC), the dorsal part of the anterior cingulate limbic cortex (dACC) along with parts of dorsal prefrontal cortex (dPFC) project to “hubs” in the medial rostral caudate nucleus, possibly to integrate computations from multiple systems (Buckner et al., 2009; Averbeck et al., 2014) that are involved in decision processes or assigning value associated with actions or stimuli (Seo et al., 2012; Averbeck et al., 2014). These “loops” appear critical for motor functions including motor control, action selection, sequence learning, and formation of habits, but also cognitive/limbic functions that include learning, memory processing, decision making, and planning (Pennartz et al., 2009; Shepherd, 2013; Saunders et al., 2015). The corticostriatal input terminates on GABAergic interneurons and MSNs. GABAergic interneurons exert *via* feed-forward inhibition on MSN and thereby exert a strong impact on MSNs, which receive both motor and non-motor inputs. This distributed input from cortical areas opens several avenues where alterations in cortical systems could lead to disrupted processing in the striatum in ASD. As one example, the dorsal anterior cingulate cortex is an area that heavily innervates the striatum (for a review see: Haber, 2016) and a recent postmortem study from our laboratory revealed differences in serotonin receptor subtype densities that were age-dependent and not evident in the other cortical brain areas examined (Brandenburg and Blatt, 2019). D1R modulates GABA release in the direct pathway whereas D2R modulates GABA release mainly to the GABAergic GPe (i.e., on striatopallidal neurons) in the indirect pathway (for a review see: Gerfen and Bolam, 2010). Recently, it was determined in the macaque that different regions of the dorsal striatum receive unique sets of cortical inputs (i.e., striatal hubs) and their striatopallidal projections are largely topographic with predictive terminal fields from individual injection sites (Heilbronner et al., 2018). Striatal zones receiving unique combinations of cortical afferents (Averbeck et al., 2014; Choi et al., 2017) parcellate into specific functional topography. An example is the convergence of projections from the inferior parietal lobule and prefrontal cortex onto the rostral dorsal caudate, which subsequently projects to the GPe and is critical for the formation of visual bias toward salient environmental stimuli (Steinmetz and Constantinidis, 1995; Corbetta and Shulman, 2002; Corbetta et al., 2008). Thus, disruption of neurochemical modulation *via* dopamine within GABAergic MSNs in the dorsal striatum has the potential to affect neuronal firing to output structures (Mamad et al., 2015), which can influence the control of specific motor and/or non-motor activity and function *via* the reciprocal output projections to cortical areas. As our results demonstrate increased expression of Drd2 and PPE mRNA, we expect that the indirect pathway striatopallidal circuity and its control by corticostriatal inputs are impacted in ASD.

To this end, there are examples of genetic autism-related animal model studies that have demonstrated changes in D2Rs with implications for BG behavioral changes that include repetitive grooming, stereotypic motor routines as well as deficits in decision making and social interactions (Fuccillo, 2016). In the mouse model of the 16p11.2 human copy number variant (CNV), there was an increase in overall numbers of D2R positive MSN phenotypes in the dorsal and ventral striatum and a strong increase in net excitatory strength on D2Rs on MSNs (Portmann et al., 2014). In another genetic animal model, Cntnap4 KOs were administered a D2R antagonist, haloperidol resulting in decreased perseverative grooming by increasing dopaminergic tone (Karayannis et al., 2014). Despite numerous examples of D2R changes in animal models, there is a paucity of studies demonstrating Drd2 mRNA changes in MSNs in animal models and in human studies. Thus, follow-up studies in postmortem autism cases are warranted to further delineate neurochemical differences within human BG circuits.

### Implications of Dysfunction of the Indirect Pathway

Disruptions in the indirect striatofugal pathway in ASD could have widespread ramifications. It may contribute to the altered encoding of information relevant to locomotion (Barbera et al., 2016). The indirect pathway projects to the GPe and dopamine controls this pathway *via* receptors expressed on striatal projection neurons or *via* presynaptic D2 receptors transported from the striatum to the Gpe (Rommelfanger and Wichmann, 2010; Mamad et al., 2015). Restricted repetitive behaviors, prevalent in ASD and other neurodevelopmental conditions, include stereotypy, rituals, and compulsions and have been reported to develop, in part, due to decreased indirect pathway activity in a mouse model (Tanimura et al., 2011). Recently, a large study of 2,084 children with ASD ≤6 years old measuring motor domain criteria from Vineland tests reported that 35% had significant motor difficulties with another 44% classified as moderately low skilled and included motor stereotypies (hand flapping, spinning, body rocking), and non-verbal behaviors such as use of body postures and gestures (Licari et al., 2020).

While many components in the brain contribute to a variety of these motor functions, MSNs in the dorsal striatum are considered to play a prominent role. The opposing actions of the direct pathway, which facilitates motor activity, vs. the indirect pathway that inhibits competing motor activity, result in smooth motor actions when relayed up to the motor cortex. If either pathway is disturbed, aberrant signals through BG efferent projections may be affected. Dopaminergic transmission dynamically regulates these actions and is critical to maintaining E/I balance within the circuitry. Since D1R excite MSNs of the direct pathway and D2 inhibits MSNs of the indirect pathway, the indirect pathway through the BG can act to terminate or inhibit competing movements selected by the direct pathway (Chu et al., 2015), i.e., if not functioning correctly, dysregulation of the specific part(s) of BG circuitry could lead to stereotypy and repetitive behaviors as seen in ASD.

### Pharmacotherapeutic Implications

Pharmacotherapies have been extensively utilized in individuals with ASD, but there are only a few FDA-approved drugs aimed at helping to ameliorate or lessen behavioral symptoms. These include Risperidone (Risperdal), a second-generation antipsychotic, which is the only drug approved by the FDA for children with ASD over 5 years of age with irritability and aggression as well as Aripiprazole (Abilify), a psychotropic drug for irritability and depression (Hirsch and Pringsheim, 2016; Li et al., 2017: Eissa et al., 2018; Lamy and Erickson, 2018). Both drugs target a combination of dopamine and serotonin receptors and both treatments have had paradoxical effects, helping some individuals with ASD but having adverse side effects in others. Each of these drugs targets BG, D2R, and 5-HT_2_. However, the present study, which is limited in cases, demonstrated similar binding densities for these key receptor types in the dorsal striatum negating any definitive conclusions regarding the efficacy of the drugs on D2R or 5-HT_2_ receptors. Future studies that address dopaminergic innervation to the GPe and possible effects on D2Rs and/or other BG structures will be important, Another consideration that cannot be ruled out is that changes in Drd2 mRNA levels are secondary to pharmacotherapy as our sample size was not suitable to assess the possible impact of treatment on mRNA levels. Earlier experimental studies in rodents and primates have provided inconsistent results on the effects of neuroleptics on mRNA levels for dopamine receptors in the BG (Fox et al., 1994). Therefore, a possible contribution of pharmacotherapy on Drd2 mRNA levels is unclear and should be further investigated.

## Conclusions

The results suggest that the indirect pathway of the BG is implicated in ASD as evidenced by a significant elevation in Drd2 mRNA within MSNs of the caudate and putamen and a similar increase in caudate MSN PPE mRNA, an indirect pathway marker. Since the indirect pathway is thought to contribute to inhibiting competing motor actions so that a chosen action by the direct pathway can proceed, it is therefore likely that a disturbance in a key regulator of action selection may be relevant for motor dysfunction, stereotypy, and/or other repetitive behaviors in individuals with ASD. In addition to these functions, the BG has been implicated in the cognitive control of language processing, and functional interactions between Broca’s area and the striatum are well documented (Bohsali and Crosson, 2016). Changes in neurochemical or cellular processes in the BG may thus also have significance to language disorders in ASD.

## Data Availability Statement

The raw data supporting the conclusions of this article will be made available by the authors, without undue reservation.

## Ethics Statement

Ethical review and approval was not required for the study on human participants in accordance with the local legislation and institutional requirements. Written informed consent for participation was not required for this study in accordance with the national legislation and the institutional requirements.

## Author Contributions

GB, J-JS and CB: conceptualization. CB, KZ, IS, BR, and MK: methodology. CB, J-JS, KZ, IS, BR, and MK: analysis. CB, GB, and J-JS: original draft. CB, GB, J-JS, KZ, IS, BR, and MK: review and editing. GB and J-JS: supervision. All authors contributed to the article and approved the submitted version.

## Conflict of Interest

The authors declare that the research was conducted in the absence of any commercial or financial relationships that could be construed as a potential conflict of interest.

## Footnotes

^1^www.zippy.nimh.nih.gov

## Abbreviations

5-HT: serotonin
ADI: Autism Diagnostic Interview
ASD: autism spectrum disorder
BG: basal ganglia
DA: dopamine
D1R: dopamine type 1 receptor
D2R: dopamine type 2 receptor
Drd2: dopamine receptor D2 gene
Drd1: dopamine receptor D1 gene
GPi: globus pallidus internus
GPe: globus pallidus externus
ISHH: *in situ* hybridization histochemistry
MSNs: medium spiny neurons
PPE: preproenkephalin
SNpr: substantia nigra pars reticularis.
